# Bidirectional feature pyramid attention-based temporal convolutional network model for motor imagery electroencephalogram classification

**DOI:** 10.3389/fnbot.2024.1343249

**Published:** 2024-01-30

**Authors:** Xinghe Xie, Liyan Chen, Shujia Qin, Fusheng Zha, Xinggang Fan

**Affiliations:** ^1^Shenzhen Academy of Robotics, Shenzhen, Guangdong Province, China; ^2^Faculty of Applied Science, Macao Polytechnic University, Macau, Macao SAR, China; ^3^Harbin Institute of Technology, Harbin, Heilongjiang Province, China; ^4^Information Engineering College, Zhijiang College of Zhejiang University of Technology, Shaoxing, China

**Keywords:** deep learning, temporal convolutional networks, multihead attention, electroencephalogram, motion imagery

## Abstract

**Introduction:**

As an interactive method gaining popularity, brain-computer interfaces (BCIs) aim to facilitate communication between the brain and external devices. Among the various research topics in BCIs, the classification of motor imagery using electroencephalography (EEG) signals has the potential to greatly improve the quality of life for people with disabilities.

**Methods:**

This technology assists them in controlling computers or other devices like prosthetic limbs, wheelchairs, and drones. However, the current performance of EEG signal decoding is not sufficient for real-world applications based on Motor Imagery EEG (MI-EEG). To address this issue, this study proposes an attention-based bidirectional feature pyramid temporal convolutional network model for the classification task of MI-EEG. The model incorporates a multi-head self-attention mechanism to weigh significant features in the MI-EEG signals. It also utilizes a temporal convolution network (TCN) to separate high-level temporal features. The signals are enhanced using the sliding-window technique, and channel and time-domain information of the MI-EEG signals is extracted through convolution.

**Results:**

Additionally, a bidirectional feature pyramid structure is employed to implement attention mechanisms across different scales and multiple frequency bands of the MI-EEG signals. The performance of our model is evaluated on the BCI Competition IV-2a dataset and the BCI Competition IV-2b dataset, and the results showed that our model outperformed the state-of-the-art baseline model, with an accuracy of 87.5 and 86.3% for the subject-dependent, respectively.

**Discussion:**

In conclusion, the BFATCNet model offers a novel approach for EEG-based motor imagery classification in BCIs, effectively capturing relevant features through attention mechanisms and temporal convolutional networks. Its superior performance on the BCI Competition IV-2a and IV-2b datasets highlights its potential for real-world applications. However, its performance on other datasets may vary, necessitating further research on data augmentation techniques and integration with multiple modalities to enhance interpretability and generalization. Additionally, reducing computational complexity for real-time applications is an important area for future work.

## 1 Introduction

The brain-computer interface (BCI) is an emerging interactive communication method that enables the neural control of prostheses and external devices, as well as post-stroke motor rehabilitation by decoding signals generated from brain activity. This state-of-the-art technology has the potential to revolutionize various aspects of life and significantly enhance the overall quality of life. BCIs have a wide range of applications, ranging from medical assistance to human enhancement (Ahmed et al., 2022; Altaheri et al., 2023). Typically, electroencephalogram (EEG) signals reflect the electrical activity of the brain and are recorded non-invasively by placing an array of electrodes on the scalp. Obtaining real values (time and channel) Two-dimensional EEG signal matrix enables direct communication between people and external devices (Graimann et al., 2010).

Motor imagery (MI) is an activity of thinking about how to move a certain part of the body without moving the body. EEG-based MI activity has been widely used in vehicle control, drone control, environmental control, smart home, security, and other non-medical fields (Altaheri et al., 2023). However, the decoding of MI-EEG signals remains a challenging task. In this task, other physiological signals, such as facial muscle activity, eye blinking, and electromagnetic interference in the environment, contaminate the recorded MI-EEG signals and result in a low signal-to-noise ratio (Lotte et al., 2018). Individual differences in MI-EEG patterns are influenced by variations in brain structure and function across participants. Additionally, the EEG system exhibits a level of correlation between signal channels, further complicating the signals processing procedure (Altaheri et al., 2022).

In traditional methods for classifying and recognizing EEG signals, there is often a reliance on domain-specific knowledge. This has led to an increased focus on developing effective feature extraction and classification techniques, primarily due to the low signal-to-noise ratio inherent in EEG signals (Huang et al., 2019). Various feature extraction methods have been commonly utilized, including independent component analysis (Barbati et al., 2004; Delorme and Makeig, 2004; Porcaro et al., 2015; Ruan et al., 2018), wavelet transform (Xu et al., 2018), common spatial pattern (Gaur et al., 2021), and empirical mode decomposition (Tang et al., 2020). After preprocessing the EEG signals, essential features are extracted from the processed signals and fed into a classifier to determine the class of input instances (Vaid et al., 2015). Traditional feature extraction methods often involve hand-designed feature extractors such as Filter Bank Shared Space Pattern (FBCSP) (Ang et al., 2008) or Riemannian Covariance (Hersche et al., 2018) features. Ang et al. (2012) used the Filter Bank Common Spatial Pattern (FBCSP) algorithm to optimize the subject-specific frequency band of Common Spatial Pattern (CSP) on MI-EEG and then employed the Mutual Information-based Best Individual Feature (MIBIF) algorithm and Mutual Information-based Rough Set Reduction (MIRSR) algorithm to extract discriminative CSP features from the signals. Finally, we use the CSP algorithm for classification and obtain good performance. It is important to note that all of these steps are computationally time-consuming.

Although traditional methods have improved the signal-to-noise ratio of EEG signals through preprocessing methods, EEG signals collected from different timestamps and subjects usually exhibit different patterns due to the inter- and intra-subject variability of the EEG signals, leading to a poor generalization of traditional methods to datasets with unknown subjects. In contrast, Deep Learning (DL) has significant advantages because it can learn complex and meaningful features directly from raw EEG signals without time-consuming preprocessing or manual feature extraction, focuses on and learns important signals from raw EEG signals, and improves the generalization of the model. DL has demonstrated remarkable success in diverse domains, such as image, video, audio, and text analysis (Hossain et al., 2018; Ahmed et al., 2019; Altaheri et al., 2019; Qamhan et al., 2021). Consequently, researchers have increasingly turned to deep learning algorithms in recent years to address EEG classification tasks, capitalizing on the significant advancements achieved by deep learning in other fields.

In recent years, there has been a surge in the use of deep learning techniques for MI-EEG classification tasks. Researchers have introduced various deep learning network models, including Convolutional Neural Networks (CNNs) (Zhang et al., 2020), Recurrent Neural Networks (RNNs) (Luo et al., 2018; Kumar et al., 2021), Deep Belief Networks (DBNs) (Xu et al., 2020), and Autoencoder (AE) structures (Hassanpour et al., 2019). Among these models, CNNs have been widely adopted, and a variety of CNN network designs have been proposed. These designs aim to learn complex and meaningful features directly from raw EEG signals, thereby improving the signal-to-noise ratio, and eliminating the need for time-consuming preprocessing or manual feature extraction. Examples of these designs include residual-based CNN (Liu and Yang, 2021), multiscale CNN (Li et al., 2020), multilayer CNN (Amin et al., 2019), and attention-based CNN (Altuwaijri et al., 2022). Bai et al. (2018) proposed a novel variant of CNNs known as temporal convolutional network for time-series modeling and classification tasks. TCN has exhibited superior performance compared to other CNNs and recurrent networks like Long Short-Term Memory (LSTM) and Gated Recurrent Units (GRU) in sequence-related tasks. The advantages of TCN are that the size of the receptive field can be expanded exponentially, the number of parameters increases linearly, and they are not affected by gradient disappearance or explosion problems. Ingolfsson et al. (2020) proposed an EEGTCN model that combines TCN and EEGNet (Lawhern et al., 2018) to maintain high classification accuracy while reducing memory footprint and computational complexity. In addition, Altaheri et al. (2022) proposed a model called ATCNet, which combines TCN, EEGNet architecture (Lawhern et al., 2018), and a multi-attention mechanism. It extracts advanced time features through TCN and EEGNet architecture, highlights the most valuable features in MI-EEG signals through the multi-attention mechanism and surpasses the performance of EEGTCN. Superior performance is achieved in subject-centered and non-subject-centered modes, respectively.

The attention mechanism is an artificial neural network structure inspired by the selective attention process of the human brain, which enables the network to focus on pertinent information. Integrating the attention mechanism into deep learning models allows for automatic learning of key features from input signals, which in combination with CNN networks can alleviate some of the limitations in MI-EEG classification, such as low signal-to-noise ratios and inter- and intra-subject variability. One of the earliest attention-based neural network models is the attention layer within the encoder-decoder framework proposed for language modeling (Hassanpour et al., 2019). The challenge lies in efficiently learning attention weights. To address this, Luong et al. (2015) introduced multiplicative attention, which further improved efficiency. The multi-head attention network, proposed by researchers at Google, further optimized attention computation (Vaswani et al., 2017). Initially, these foundational attention models were applied in the field of natural language processing (NLP) and achieved success. Subsequently, they were extended to the domain of computer vision. Attention mechanisms proposed for the visual domain include squeeze-and-excitation blocks (Hu et al., 2018) and convolutional block attention modules (CBAMs) (Woo et al., 2018). These mechanisms facilitate the network in learning the correlations between different time steps and channels, thereby enhancing its ability to capture relevant visual information. Zhang et al. (2020) proposed a Graph-based Convolutional Recurrent Attention Model (G-CRAM). The model buildings a graph structure to represent the positioning information of EEG and employs a convolutional recurrent attention mechanism to learn spatial and temporal EEG features, with a focus on the most discriminative temporal periods, which overcomes the challenges of complexity, dynamics, and low signal-to-noise ratio of the EEG signals, and obtains superior performance in the MI-EEG classification task. Altuwaijri et al. (2022) proposed a novel model called Multi-Branch EEGNet with squeeze-and-excitation blocks (MBEEGSE) for decoding EEG-based motor imagery. The model aims to overcome the challenges of inter-subject and intra-subject variability of EEG signal and low signal-to-noise ratio to extract high-level features of EEG signal. The model employs a multi-branch convolutional neural network architecture with attention blocks to capture channel interdependencies and adaptively modify channel-wise feature responses. Superior performance is obtained in MI-EEG classification tasks.

In the future, MI-EEG classification tasks could benefit from research on artificial general intelligence methods to achieve high levels of intelligence, high precision, high robustness, and low power consumption. In this regard, Yang et al. put forward a series of innovative methods. First, Yang et al. (2022) propose a novel spike-based framework with minimum error entropy, called MeMEE, The framework combines entropy theory and recurrent spiking neural network (SNN) architecture and establishes a gradient-based online meta-learning scheme to improve the accuracy and robustness of SNN in various tasks. Second, Yang and Chen (2023) propose a novel and flexible learning framework termed high-order spike-based information bottleneck (HOSIB) leveraging the surrogate gradient technique for peak-based machine intelligence. The framework utilizes the surrogate gradient technique second-order information bottleneck (SOIB) and third-order information bottleneck (TOIB) to explore the underlying architecture and peak-based intrinsic information in SNN models. By discarding redundant information, the HOSIB framework improves the generalization and robustness of SNN models. Experiments show that the framework has superior generalization ability, robustness, and power efficiency. In addition, Yang et al. (2023) proposes an efficient learning mechanism for spiking dendrites, addressing the challenge of designing efficient learning mechanisms with dendrites. The method utilizes a multi-scale learning rule with dendritic predictive characteristics and employs a two-phase learning mechanism based on burst-related plateau potential dynamics of spiking dendrites. The experimental results have demonstrated that the proposed algorithm improves learning accuracy and reduces synaptic operations. This reduction in synaptic operations and spike numbers in the output layer leads to a reduction of power consumption on neuromorphic hardware. The combination of the three-factor dendritic prediction principle and two-phase plateau potential activities enhances learning capability and sparsity within a single neuron, while also improving robustness and learning convergence speed.

This study presents a novel bidirectional feature pyramid network attention-based temporal convolutional network, BFATCNet, for decoding MI-EEG brain signals. To enhance the input signals, data augmentation techniques such as data blending, Gaussian noise addition, and signal scaling are employed. The proposed BFATCNet model follows a four-stage process for processing MI-EEG signals. First, the MI-EEG signal undergoes the encoding stage using a combination of CNN, CBAM, and Bidirectional Feature Pyramid Network (Bi-FPN) to generate a series of high-level temporal representations. This stage aims to capture the correlations between different channels and time steps, resulting in time series signals for various frequency bands. Second, an attention layer is utilized to highlight the most salient information within the time series of different frequency bands. This attention mechanism assists the model in focusing on significant features and enhancing the discriminative power of the network. Third, a temporal convolutional layer is employed to extract high-level temporal features from the attention-highlighted information. This layer leverages temporal relationships in the signals to capture important patterns and dynamics. Finally, a fully connected layer analyzes the extracted high-level temporal features for classification purposes. This study makes several significant contributions:

1. The proposed BFATCNet model integrates the mechanisms of TCN, CBAM, Bi-FPN, attention, and convolution-based sliding window to achieve state-of-the-art performance in the BCI Contest IV-2a dataset.
2. The incorporation of CBAM facilitates the model's ability to capture correlations between different channels and time steps in the signals. Moreover, the multi-head attention mechanism enhances the model's focus on important MI information within the MI-EEG signals, allowing for effective learning and utilization of essential patterns and relationships.
3. The utilization of the Bi-FPN structure addresses the limitation of previous models that only focus on a single frequency band of EEG. By considering the information from the time series of different frequency bands, the model can improve its performance by leveraging the diverse and complementary information available across various frequency bands. This demonstrates the effectiveness of the Bi-FPN structure in enhancing the model's understanding of the MI-EEG signals.

## 2 Design of the BFATCNet model

**Figure 1:**
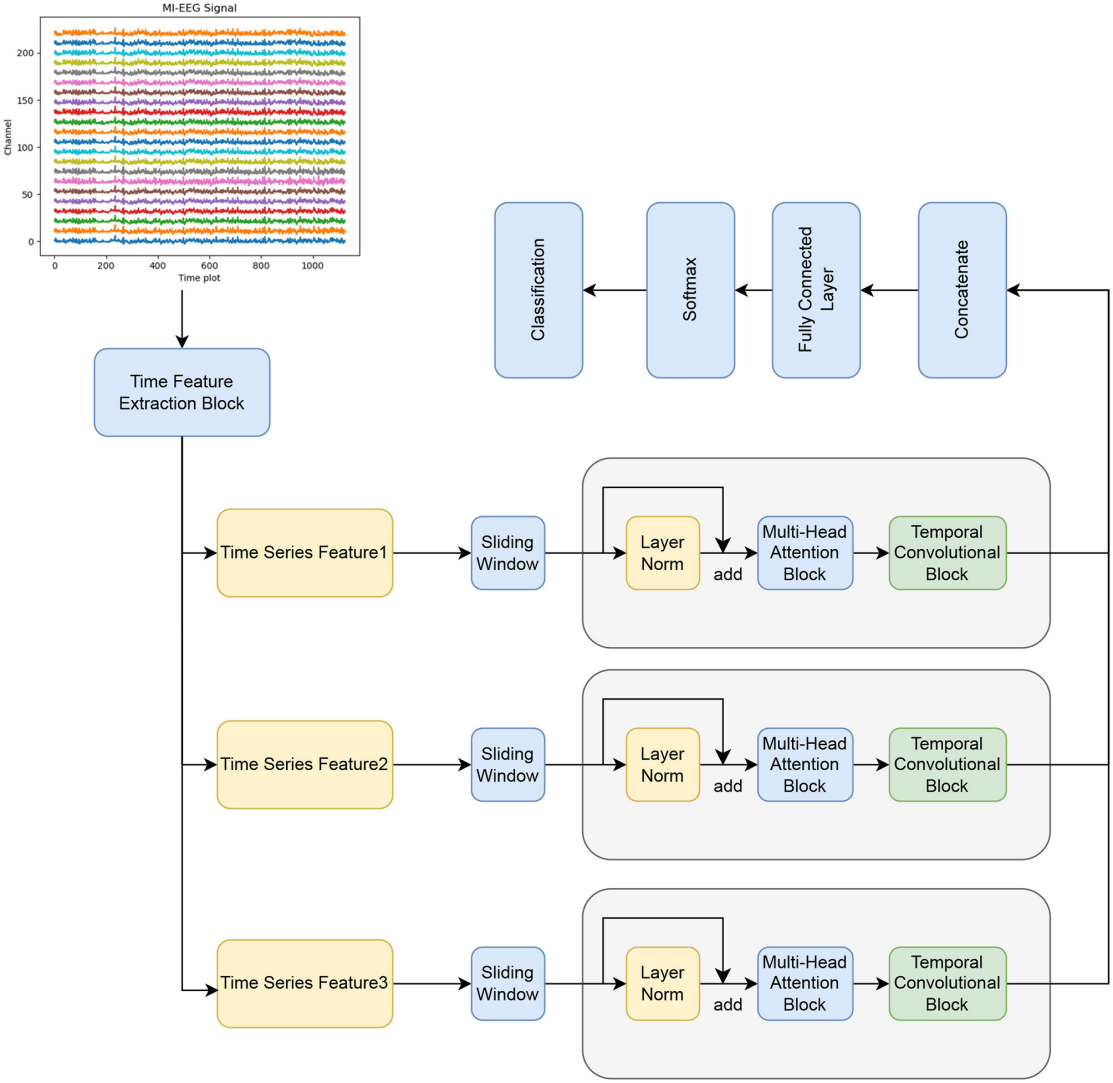
The components of the BFATCNet model.

The proposed BFATCNet model consists of four main blocks: temporal feature block, attention (AT) block, and temporal convolution (TC) block with full connectivity, as shown in Figure 1. The temporal feature block encodes the original MI-EEG signals using temporal convolution layers, which include temporal convolution, channel depth convolution, and spatial convolution. It also incorporates the channel attention mechanism CBAM and the Bi-FPN structure. The block learns the correlation between channels and different time steps, extracts low-level temporal feature representations for different frequency bands and time steps, and resolves the effects of inter- and intra-subject variability and low signal-to-noise ratios of EEG signals on the classification performance of the model. Next, the AT block utilizes the Multiple Self-Attention (MSA) mechanisms to emphasize the components of the time series that have the highest correlation between different features, enhancing the generalization of the model to unknown subject datasets. Finally, the TC block applies TCN to extract high-level temporal features in the time series. The temporal features from different frequency bands are then concatenated and fed into the fully connected block for classification and identification.

The output of the time series generated by the temporal feature block can be divided into multiple windows. Each window is separately processed by the AT/TC block. The outputs of all windows are then concatenated and passed through the softmax classifier. This approach enhances data efficiency and improves accuracy. More information about the BFATCNet block is provided in the subsequent subsections.

### 2.1 Temporal feature block

The temporal feature block is based on the EEGNet architecture originally proposed by Lawhern et al. (2018), but with modifications. Unlike the original design that uses separable convolution, the temporal feature block utilizes 2D convolution, which has shown enhanced performance. Additionally, this block incorporates the CBAM attention mechanism to capture correlations between channels and different time steps. Moreover, the Bi-FPN architecture is employed to obtain representations of the time series in various frequency bands. The temporal feature block is used to extract high-dimensional features of different bands in EEG signals, and the CBAM attention mechanism is used to capture the correlation between channels and different time steps, which improves the generalization of the model.

**Figure 2:**
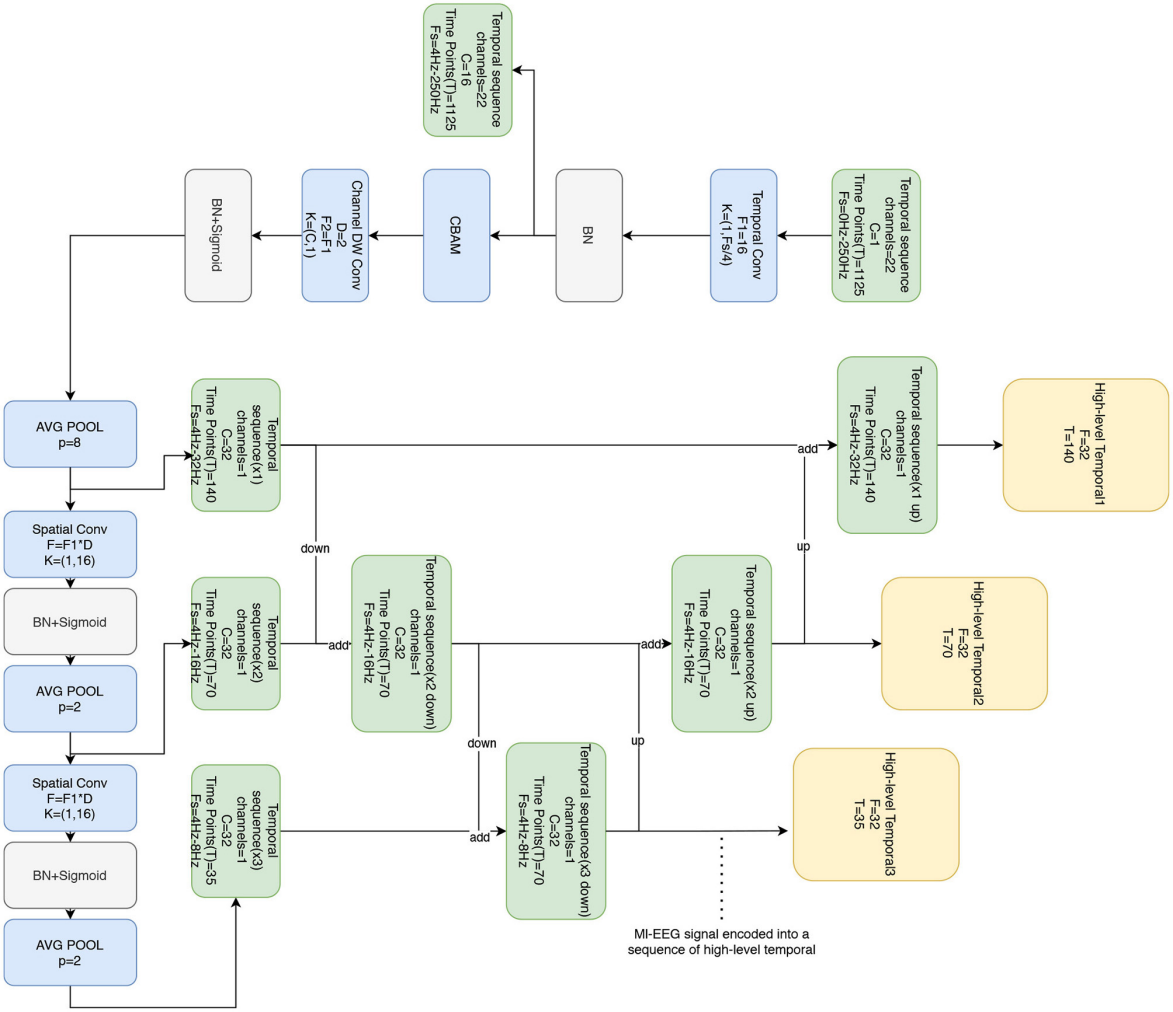
Temporal feature block consists of four convolutional layers and CBAM attention mechanism, which receives the original MI-EEG signals and outputs the temporal signals in different frequency bands.

The temporal feature block consists of four convolution (conv) layers and the CBAM attention mechanism, as shown in Figure 2. Firstly, a temporal convolution is applied using F1 filters with a size of (1, *F*_*s*_/4), where *F*_*s*_/4 represents the length of the filter along the time axis. In the BCI-2a dataset, which has a sampling rate of 250 Hz, *F*_*s*_/4 becomes 62.5. To conform to standard lengths, the closest value, 64, is selected. This choice ensures the extraction of temporal information associated with frequencies above 4 Hz. The output of this layer corresponds to the F1 temporal feature maps. This design facilitates the extraction of temporal information related to higher frequencies within the time series signals, enabling the capture of subtle changes and dynamic features present in the signals.

**Figure 3:**
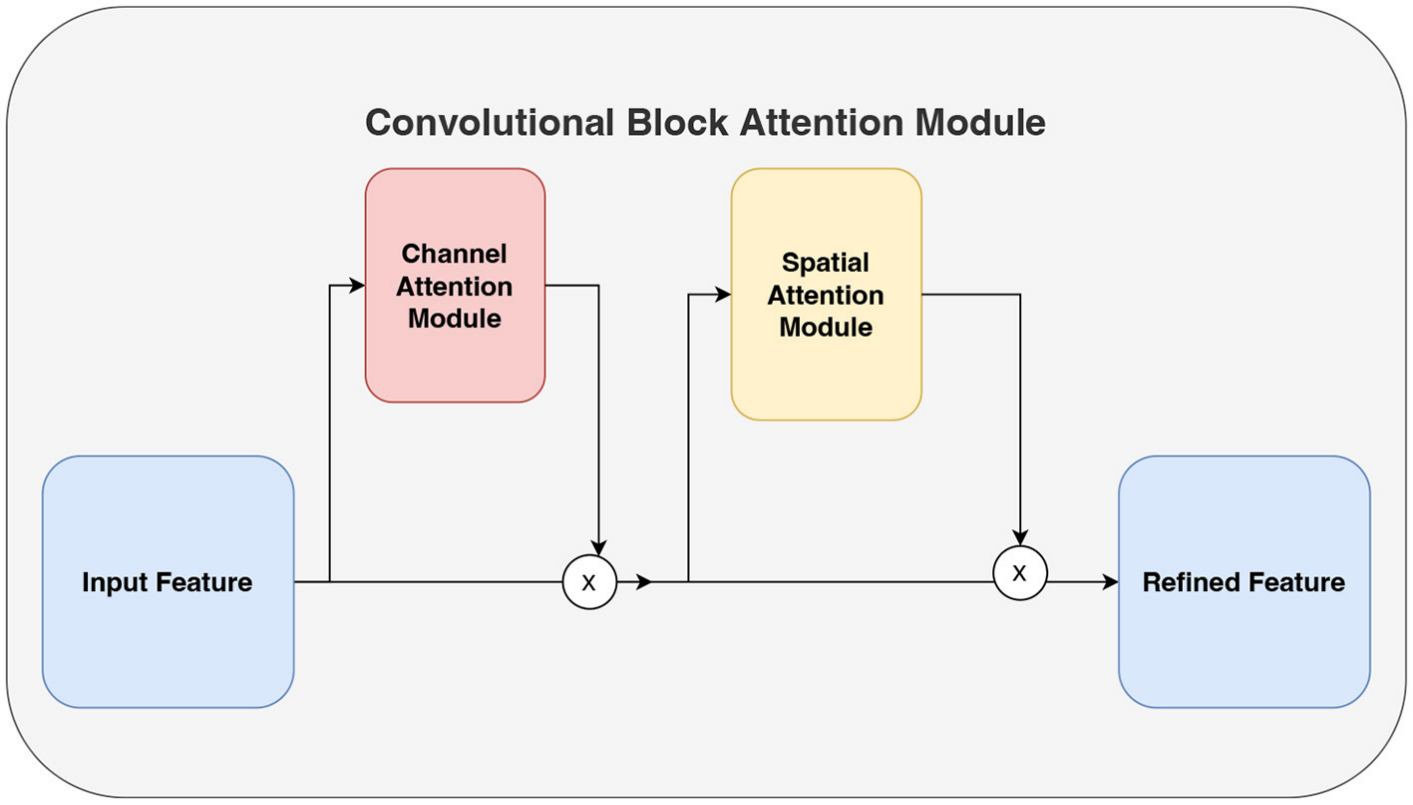
CBAM attention mechanism consisting of channel attention and spatial attention.

Convolutional block attention module (CBAM) (Woo et al., 2018) comprises both channel attention and spatial attention mechanisms as shown in Figure 3. The channel attention mechanism focuses on extracting significant information from the input signals along the channel dimension. It consists of three main components: the Adaptive Average Pooling Layer (AdaptiveAvgPool2d), the Adaptive Maximum Pooling Layer (AdaptiveMaxPool2d), and the Shared Multi-Layer Perceptron (SharedMLP). Initially, the input signals undergo pooling operations using the adaptive average pooling layer and adaptive maximum pooling layer, resulting in the average pooled output and maximum pooled output, respectively. Subsequently, the shared multi-layer perceptron applies a convolution operation to these outputs, allowing the extraction of feature representations within the channel dimensions. Finally, the convolution output is activated by a sigmoid function, producing the channel attention weights. These weights are then used to emphasize important channel information by appropriately weighting the different channels of the input signals.

On the other hand, the spatial attention mechanism aims to extract significant information from the input signals along the spatial dimension. It consists of a convolutional layer (Conv2d) that is subsequently followed by a sigmoid activation function. The input to this convolutional layer is the data that has been processed by the channel attention mechanism, resulting in a two-channel input. After the convolution operation, the output is activated by the sigmoid function, producing spatial attention weights. These weights are used to highlight important spatial information by assigning different weights to different spatial locations of the input signals accordingly. The integration of the channel attention mechanism and the spatial attention mechanism enables the model to learn correlations between channels and different time steps, thereby enhancing its understanding of the signals.

The second layer utilizes deep convolution with F2 filters of size (C, 1), where C denotes the number of EEG channels. This deep convolution allows each filter to extract spatial features, particularly features related to the EEG channels, from a single temporal feature map. As a result, the output of this layer consists of F2×*D* feature maps, where *D* represents the number of filters associated with each temporal feature map in the previous layer. Based on practical experience and signal characteristics, the value for D is determined as 2.

After the deep convolution layer, an average pooling layer with a size of (1, 8) is utilized to achieve an eight-fold abstraction of the temporal signals. This pooling operation reduces the signal's sampling rate to approximately 32 Hz. The rationale behind this design choice is to improve the extraction of spatial features and abstract the signal. By enabling more effective feature extraction and dimensionality reduction of the signals, this approach enhances the overall performance of the model.

The third and fourth layers involve spatial convolutions using F2 × *D* filters with a size of (1, 16) to perform spatial convolutions. The filter length along the time axis is 16, and these convolutions are applied to decode the 4–32 Hz motor imagery (MI) activity and 4–16 Hz activities, respectively. To decrease the sampling rate and adjust the length of the resulting time series, an average pooling layer with a size of (1, 2) is used. Additionally, batch normalization is implemented to expedite network training. The nonlinear activation function applied in these layers is the sigmoid activation function.

The Bi-FPN is critical in improving the model's performance by effectively integrating feature map information from different layers. In this case, the time series outputs from convolutional layers 2, 3, and 4 are fed into the Bi-FPN. As a result, integrated outputs for layers 2, 3, and 4 are obtained, encompassing the 4–32, 4–16, and 4–8 Hz time series across three distinct frequency bands.

The temporal feature block outputs three-time series $z_{j}\in {\mathbb{R}}^{T_{c}\times d}$ in three different frequency bands consisting of time vectors *T*_*c*_ of 140, 70, and 35 respectively. We empirically set *d* to 32. The length of the time series *z*_*j*_ is determined by $T_{c}=\frac{T}{P}$, where *T* refers to the time point of the original EEG signal, and *P* is the cumulative multiplication of the kernel of the pooling layer that has passed through.

### 2.2 Sliding window

To capture the dynamic properties and timing patterns of the signals more effectively, a sliding window approach is employed instead of directly inputting the entire *z*_*j*_ into subsequent layers (Schirrmeister et al., 2017). By utilizing a sliding window of length *T*_*w*_, the time series *z*_*j*_ is systematically divided into multiple windows denoted as $z_{j}^{w}\in {\mathbb{R}}^{T_{w}\times d}$, where *w* represents the window index ranging from 1 to *n*, the total number of windows. Subsequently, each window $z_{j}^{w}$ is individually processed by the subsequent Attention block and Temporal Convolutional block. According to Equation (1), a specific value for the window length *T*_*w*_ can be calculated in the following way:


(1) $$\begin{array}{l} T_{w}=T_{c}-n+1,\;T_{c}>n\geq 1 \end{array}$$

Suppose the temporal feature block utilizes three pooling layers with respective sizes of *P*_1_ = 8, *P*_2_ = 2, and *P*_3_ = 2. In that case, it generates three time series *z*_1*i*_, *z*_2*i*_, and *z*_3*i*_ comprising three vectors of size *T*_1_ = 140, *T*_2_ = 70, and *T*_3_ = 35, respectively. Each of these time series represents 32 (= 8 × 2 × 2), 16 (= 8 × 2), and 8-time points, respectively, from the original MI-EEG signal *x*. Therefore, one step of sliding in *z*_1*i*_, *z*_2*i*_, and *z*_3*i*_ corresponds to 32, 16, and 8 steps of sliding in the original signal *x*.

### 2.3 Attention (AT) block

The attention mechanism, introduced by Vaswani et al. (2017), is a neural network structure that emulates the selective information-focusing behavior observed in the human brain. By integrating the attention mechanism into deep learning models, it becomes possible to automatically extract essential information from the input signals. Improving the generalization of the model. An important feature of the multi-head attention mechanism is the internal variability, which allows the model to learn different attention weights among different heads, thus further improving the generalization performance. Following the division of the time series into multiple segments through the sliding window approach, *N* segments of $e_{j}^{i}$, where *i* represents the *i*th head and *j* denotes the *j*th vector of *e*^*i*^, are created, corresponding to the number of heads *N* in the multi-head attention mechanism. Each instance of the time series, denoted as $e_{j}^{i}$, is then multiplied by *W*_*q*_, *W*_*k*_, and *W*_*v*_ to derive the respective query vector *q*_*j*_, key vector *k*_*j*_, and value vector *v*_*j*_ as shown in Equations 2–4:


(2) $$\begin{array}{ll} q_{j} & =e_{j}^{i}\cdot W_{q} \end{array}$$

(3) $$\begin{array}{ll} k_{j} & =e_{j}^{i}\cdot W_{k} \end{array}$$

(4) $$\begin{array}{ll} v_{j} & =e_{j}^{i}\cdot W_{v} \end{array}$$

The attention value is computed based on the query vector $q_{j}^{i}$, key vector $k_{j}^{i}$, and value vector $v_{j}^{i}$. To calculate the normalized correlation score between the *a*th coding vector $e_{a}^{i}$ and the *b*th coding vector $e_{b}^{i}$, as shown in Equation 5


(5) $$\begin{array}{l} s_{a}^{i}=\text{softmax}\left(\frac{q_{a}^{i}{\left(k_{b}^{i}\right)}^{T}}{\sqrt{d_{k}}}\right) \end{array}$$

where *d*_*k*_ is the dimension of $k_{b}^{i}$. Then, the attention-weighted output $z_{a}^{i}$ is defined as Equation 6


(6) $$\begin{array}{l} z_{a}^{i}=\overset{T}{\underset{b=1}{\sum }}s_{a}^{i}v_{b}^{i},\;a\in \left\{1,2,\ldots ,T\right\} \end{array}$$

where *T* is the number of rows of the *v*^*i*^ matrix set to coincide with the time length of the time series output from the temporal series block empirically. Finally, *z*^*i*^s are spliced as *Z* = [*z*^1^, *z*^2^, ..., *z*^8^] to obtain the time series after highlighting the important information.

### 2.4 Temporal convolutional (TC) block

**Figure 4:**
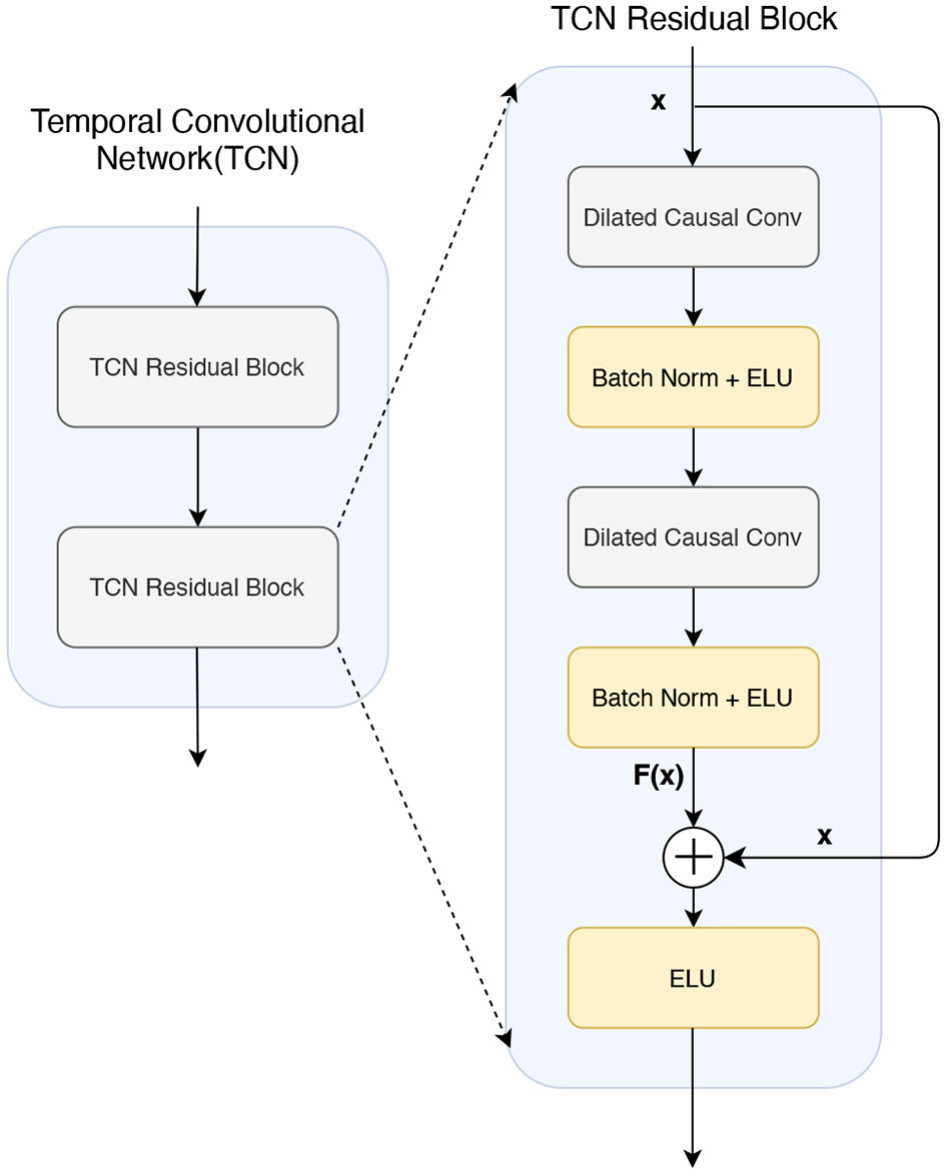
The architecture of a temporal convolutional network (TCN) consists of two residual blocks.

TCN architecture is composed of multiple residual blocks. Each residual block comprises two dilated causal convolution layers (Ingolfsson et al., 2020), followed by batch normalization and Exponential Linear Unit (ELU) activation, as depicted in Figure 4. The adoption of dilated causal convolution exponentially extends the receptive field, ensuring that no information propagates from future time steps to past time steps. Therefore, the output of time *t*is completely dependent on the input of time *t*or before, so that the relationship in the long series can be better learned, ensuring that the model is invariant to the translation of the time series, that is, robust to the translation of the signal in time. The residual block employed in the TCN performs element-level summation, denoted as *F*(*x*)+*x*, on the input and output feature maps. This summation aids in learning constant functions and prevents the vanishing or exploding of gradients in the model. With residual blocks, the model can be sensitive to translation while learning local changes and global trends in the time series data. When the signal shifts in time, residual blocks can help the model adapt better to this change, thus improving translation invariance. Within the residual blocks, a constant mapping strategy is employed, resulting in an exponential increase in the receptive field size (RFS) of the TCN with the number of stacked residual blocks *L*. This increase is attributed to the exponential expansion *D* observed in each subsequent block. The RFS is computed as in Equation 7 and is determined by two key parameters: the number of remaining blocks *L* and the convolutional kernel size *K*_*T*_.


(7) $$\begin{array}{l} \text{RFS}=1+2\left(K_{T}-1\right)\left(2^{L}-1\right) \end{array}$$

A typical configuration of the TC block in BFATCNet consists of *L* = 2 residual blocks and 32 filters of size *K*_*T*_ = 4 for all convolutional layers, so RFS is 19. With this setting, the TCN can process up to 19 elements in a sequence.

### 2.5 Fully connected block

In the final stage of the proposed model, three groups of time series with distinct frequency bands undergo sliding window, AT block, and TCN processing. Following the derivation of advanced time features, Adaptive Average Pooling is implemented on the three groups of advanced time features to compress them into predetermined features. The flattened advanced time features are then concatenated and subsequently passed through a three-layer fully connected layer. To expedite network training, batching is employed in combination with the fully connected layers. Additionally, Dropout is utilized to mitigate overfitting. Finally, the resulting outputs are fed into a softmax function for probability computation. The hyperparameters of the model are determined empirically and further tuned using Optuna (Akiba et al., 2019). The specific values of these hyperparameters are as follows: for the AT block, two attention heads are used with a head size of 32. For the TC block, two residual blocks are utilized with a kernel size of 4 and a total of 32 filters. The dropout rate for both AT blocks and TC blocks is set to 0.12.

## 3 Experimental results and discussion

### 3.1 Datasets and data enhancement

The BCI Contest IV-2a (BCI-2a) dataset (Brunner et al., 2008) is a widely recognized publicly available dataset for Motor Imagery Electroencephalography (MI-EEG) analysis. This dataset serves as a benchmark for MI-EEG decoding research. The BCI-2a dataset includes EEG data recorded from nine subjects, with 22 channels sampled at a rate of 250 Hz. During the data collection process, participants were given instructions to perform four different motor imagery tasks: left-hand movement, right-hand movement, foot movement, and tongue movement. Two sessions were conducted for each subject on separate days, resulting in a total of 288 trials per session. In each trial, participants performed motor imagery from the interval of 2s to 6s. It's important to note that only one session in the dataset contains class labels for all trials, whereas the other session was used as the target domain.

The BCI Contest IV-2b (BCI-2b) dataset (Leeb et al., 2008) is a widely recognized publicly available dataset for Motor Imagery Electroencephalography (MI-EEG) analysis. This dataset serves as a benchmark for MI-EEG decoding research. In the BCI-2b dataset, EEG data from three channels (C3, Cz, and C4) were captured at a sampling rate of 250 Hz from nine subjects. During the experiments, after the cue appeared, all subjects were instructed to imagine left or right-hand movements for four seconds. Each subject was provided with five sessions, with each session consisting of 120 trials. The first three sessions in the dataset were well-labeled, while the last two sessions were not. In the conducted experiments, the first three sessions were treated as source domains, and the remaining two sessions were considered as target domains. Additionally, since the first three sessions were collected at different times, they naturally represent three distinct source domains.

The proposed model is evaluated using subject-dependent (subject-specific). The model is trained and tested based on the data of individual subjects. Data augmentation was applied to the BCI-2a dataset and BCI-2b dataset to enhance the model's generalization capabilities and improve noise robustness. The augmentation involved mixing the signals from two different samples with the same label, adding Gaussian noise *n*, and scaling the signal using the formula where *w* ∈ (0, 1), noise level ∈ (0, 0.3), and scale ∈(0.8, 1.2), as shown in Equations (8, 9, 10).

To assess the impact of the data enhancement process on model performance, we evaluated the performance of two models: accuracy and kappa, on the BCI-2a dataset and the BCI-2b dataset. We compared the performance of the model using data enhancement with that of the model without data enhancement. The results show that on the BCI-2a dataset, the overall accuracy of the model using data augmentation increased by 13.3% and kappa increased by 0.17. On the BCI-2b dataset, the overall accuracy of the model using data augmentation increased by 4.5% and kappa increased by 0.08. The results show that data augmentation improves the performance of the model on different datasets with a certain effect, especially the performance improvement on the BCI-2a dataset is more significant.


(8) $$\begin{array}{ll} x_{h} & \leftarrow w\cdot x_{1}+\left(1-w\right)\cdot x_{2} \end{array}$$

(9) $$\begin{array}{ll} x_{h} & \leftarrow x_{h}+\text{noise level}\cdot n \end{array}$$

(10) $$\begin{array}{ll} x_{h} & \leftarrow \text{scale}\cdot x_{h} \end{array}$$

### 3.2 Performance metrics

In this study, the model performance was assessed using the following methods Accuracy (Acc), Equation 11 and κ score, Equation 12.


(11) $$\begin{array}{l} \text{Acc}=\frac{1}{n}\overset{n}{\underset{i=1}{\sum }}\frac{{\text{TP}}_{i}}{H_{i}} \end{array}$$

where TP_*i*_ is the true positives, *H*_*i*_ is the number of samples in class *i*, and *n* denotes the number of classes;


(12) $$\begin{array}{l} \kappa =\frac{1}{n}\overset{n}{\underset{a=1}{\sum }}\frac{P_{a}-P_{e}}{1-P_{e}} \end{array}$$

where *P*_*a*_ is the actual percent agreement, and *P*_*e*_ is the expected percent agreement probability (Cohen, 1960).

### 3.3 Training procedure

The models in this study were trained and evaluated using the PyTorch framework. A consistent configuration was followed for the training process. The model parameters were initialized with weights drawn from a normal distribution with a mean of 0 and a standard deviation of 0.01. The AdamW optimizer was used for training the models, with a learning rate of 1.795 × 10^−3^ and a weight decay rate of 5.015 × 10^−8^. A batch size of 128 was utilized, and the training was conducted for more than 40 epochs. The categorical cross-entropy loss function was used as the objective function during training. To mitigate overfitting, a dropout rate of 0.12 was applied. These hyper-parameters were determined through a series of experiments, coupled with Optuna tuning, to ensure optimal generalization of the model. The proposed BFATCNet model achieved an impressive overall accuracy of 87.5% and a κ score of 0.83, surpassing the state-of-the-art performance in this domain.

### 3.4 Ablation study

Table 1 presents the impact of removing one or more blocks from the BFATCNet model on MI classification performance, using the BCI-2a dataset. The blocks were removed before training and validation procedures. The results demonstrate that the Bi-FPN blocks contribute significantly to the overall accuracy of the model, improving it by 8%, while the CBAM block improves the accuracy by 2.4%. The combination of Bi-FPN and CBAM blocks leads to an overall accuracy improvement of 8.7%. Table 2 presents the impact of removing one or more blocks from the BFATCNet model on MI classification performance, using the BCI-2b dataset. The blocks were removed before training and validation procedures. The results demonstrate that the Bi-FPN blocks contribute significantly to the overall accuracy of the model, improving it by 10%, while the CBAM block improves the accuracy by 2.8%. The combination of Bi-FPN and CBAM blocks leads to an overall accuracy improvement of 11.5%. These findings underscore the pivotal role of the Bi-FPN block in the BFATCNet model, primarily through the acquisition of multiple time series featuring distinct frequency bands. Additionally, the incorporation of the CBAM block also contributes to improving performance. Notably, the combination of Bi-FPN and CBAM blocks results in a synergistic effect that amplifies the benefits of each block, leading to a greater improvement in the overall accuracy of the model.

**Table 1:** The contribution of each block in BFATCNet was evaluated using the BCI-2a dataset.

**Removed block**	**Acc**	**κ**
None (BFATCNet)	87.5%	0.83
Bi-FPN	79.5%	0.72
CBAM	85.1%	0.80
Bi-FPN + CBAM	78.8%	0.71

**Table 2:** The contribution of each block in BFATCNet was evaluated using the BCI-2b dataset.

**Removed block**	**Acc**	**κ**
None (BFATCNet)	86.3%	0.73
Bi-FPN	76.3%	0.52
CBAM	83.5%	0.67
Bi-FPN + CBAM	74.8%	0.49

### 3.5 Comparison with recent studies

Table 3 presents a comprehensive summary of the accuracy and kappa scores achieved by the BFATCNet model and its comparison model on the BCI-2a dataset for different subjects. The results demonstrate that the BFATCNet model, the ATCNet model, and the TSCT model exhibit the capability to learn distinct attention weights based on the EEG signals from different subjects, utilizing the multi-head attention mechanism to enhance generalization performance. They have shown superior performance compared to other models in terms of average accuracy and standard deviation of accuracy. The BFATCNet model, in particular, leverages the features of different frequency bands in the EEG signals, resulting in further improved model performance. It outperforms the other models with an average accuracy of 87.5% and a kappa score of 0.83. Table 4 presents a comprehensive summary of the average accuracy and kappa scores achieved by the BFATCNet model and its comparison model on the BCI-2b dataset for different subjects. A comparison is made with other similar models: DRDA, DAFS, DAWD, GAT, and DJDAN. The results clearly show that the GAT model uses an attention-based domain adaptation approach for capturing globally correlated features between the source and target domains to address inter- and intra-subject variability of EEG signals and to enhance the generalization performance. The BFATCNet model learns different attention weights based on EEG signals from different subjects through the multi-attention mechanism algorithm, which enhances the generalization performance. It outperforms the other models with an average accuracy of 86.3% and a kappa score of 0.72.

**Table 3:** Comparison of the performance between the proposed model and other replicated models for topic-specific classification using the BCI-2a dataset.

**Subject**	**EEG-TCNet**	**EEGNet**	**TCNet**	**TSCT**	**ATCNet**	**BFATCNet (ours)**
	**Acc (%)**	**Acc (%)**	**Acc (%)**	**Acc (%)**	**Acc (%)**	**Acc (%)**
A01	84.0	88.5	86.1	87.9	**88.5**	82.1
A02	66.3	66.0	66.0	71.9	70.5	**86.3**
A03	94.1	95.1	93.4	95.8	**97.6**	91.2
A04	72.6	73.6	72.6	84.0	81.0	**86.1**
A05	76.0	75.4	79.9	78.1	83.0	**96.0**
A06	62.9	64.2	66.7	67.7	73.6	**89.3**
A07	89.9	90.3	90.3	91.0	**93.1**	89.0
A08	84.7	85.8	85.8	85.1	**90.3**	85.1
A09	85.4	86.5	85.4	88.2	**91.0**	83.2
MEAN (Acc)	79.6	80.6	80.7	83.3	85.4	87.5
STD (Acc)	10.7	11.1	10.1	8.59	9.1	4.4
Kappa	0.73	0.74	0.74	N/A	0.81	0.83

The bold values indicate the performance of the model that performs best among all the models compared in a single subject, while bold underlined values indicate the performance of the model that performs best among all the models compared in all the subjects.

**Table 4:** Comparison of the performance between the proposed model and other replicated models for topic-specific classification using the BCI-2b dataset.

**Subject**	**DRDA**	**GAT**	**DJDAN**	**DAFS**	**DAWD**	**BFATCNet (ours)**
	**Acc (%)**	**Acc (%)**	**Acc (%)**	**Acc (%)**	**Acc (%)**	**Acc (%)**
A01	81.4	84.6	75.8	70.3	**84.6**	84.2
A02	62.9	61.7	58.5	73.5	66.6	**77.1**
A03	63.6	60.8	73.0	80.3	68.0	**89.1**
A04	95.9	**99.6**	96.7	94.7	96.8	97.5
A05	93.6	87.5	98.9	**95.0**	94.3	87.7
A06	88.2	**93.3**	87.6	83.7	82.6	85.4
A07	85.0	85.4	85.7	**93.7**	88.4	84.1
A08	**95.2**	95.0	84.3	95.0	93.9	89.1
A09	90.0	**92.0**	85.3	75.3	90.1	82.9
MEAN(Acc)	83.9	84.4	84.6	84.6	85.0	86.3
STD(Acc)	12.6	13.1	12.3	9.6	10.4	5.2
Kappa	0.67	0.68	0.69	0.69	0.70	0.72

The bold values indicate the performance of the model that performs best among all the models compared in a single subject, while bold underlined values indicate the performance of the model that performs best among all the models compared in all the subjects.

It is worth noting that the standard deviation of BFATCNet's performance is only 4.4% across subjects in the BCI-2a dataset and 5.2% across subjects in the BCI-2b dataset, which suggests that it has a high degree of stability in its classification effect across individuals. In addition, the performance consistency of the BFATCNet model among different users is also improved. In addition, the BFATCNet model shows higher performance consistency among different users.

According to the results in Tables 5, 6, the BFATCNet model outperformed the other models in decoding all MI categories in a subject-specific motor imagery (MI) categorization study. In particular, the BFATCNet model showed higher overall accuracy and kappa scores in a per-subject MI-EEG classification task compared to recent studies using raw EEG signals. These findings suggest that the BFATCNet model learns different attention weights for different subjects' EEG signals through a multi-head attention mechanism that adapts to the individual differences of different subjects, which enables the BFATCNet model to better decode the MI task for a specific subject, showing higher performance and accuracy compared to other models.

**Table 5:** Classification performance of different methods on BCI-2a dataset.

**Method**	**Acc (%)**	**κ**
Shallow CNN (Schirrmeister et al., 2017)	74.31	0.66
EEGNet: CNN (Lawhern et al., 2018)	80.59	0.74
DBN-AE (Hossain et al., 2018)	71.00	N/A
Multi-layer-CNN and MLP (Amin et al., 2019)	75.00	N/A
EEG-TCNet: CNN and TCN (Ingolfsson et al., 2020)	79.55	0.73
Attention multi-scale CNN (Li et al., 2020)	79.90	N/A
TCNet fusion: multi-layer CNN + TCN (Musallam et al., 2021)	80.67	0.74
Attention multi-branch CNN (Altuwaijri et al., 2022)	82.84	0.77
ATCNet: attention-CNN and TCN (Altaheri et al., 2022)	85.38	0.80
TSCT: temporal-spatial convolution and transformer (Shi et al., 2023)	83.3	N/A
BFATCNet: Bi-FPN attention-CNN and TCN (proposed)	87.50	0.83

The bold values indicate the performance of the model that performs best among all the models compared in a single subject, while bold underlined values indicate the performance of the model that performs best among all the models compared in all the subjects.

**Table 6:** Classification performance of different methods on BCI-2b dataset.

**Method**	**Acc (%)**	**κ**
FBCSP: filter bank common spatial pattern (Ang et al., 2008)	80.00	0.60
EEGNet: CNN (Lawhern et al., 2018)	82.37	0.65
CCSP: composite common spatial pattern (Kang et al., 2009)	72.70	0.45
ConvNet: CNN (Schirrmeister et al., 2017)	79.37	0.58
DRDA: deep representation-based domain adaptation (Zhao et al., 2020)	83.98	0.67
DJDAN: dynamic joint domain adaptation network (Hong et al., 2021)	84.66	0.69
DAFS (Phunruangsakao et al., 2022)	84.63	0.69
DAWD: domain adaptation network based on Wasserstein distance (She et al., 2023)	85.06	0.70
GAT: global adaptive transformer (Song et al., 2023)	84.44	0.68
BFATCNet: Bi-FPN attention-CNN and TCN (proposed)	86.38	0.72

The bold values indicate the performance of the model that performs best among all the models compared in a single subject, while bold underlined values indicate the performance of the model that performs best among all the models compared in all the subjects.

## 4 Conclusion

The present study proposes a novel attention-based bidirectional Feature Pyramid Network temporal convolution network (BFATCNet) for EEG-based motor imagery classification. BFATCNet comprises four key blocks: a temporal feature block, an attention (AT) block, a temporal convolution (TC) block, and a fully connected block. The temporal feature block encodes the raw MI-EEG signals using a temporal convolutional layer, a channel attention mechanism CBAM, and a Bi-FPN structure. The low-level temporal feature representations of different frequency bands and time steps are extracted to learn the correlation between cross-channel signals and different time steps and to address the impact of inter- and intra-subject variability and low signal-to-noise ratio of EEG signals on the classification performance of the model. Second, the AT block uses the Multihead Self-Attention mechanism to learn different attention weights based on the EEG signals from different subjects to emphasize the components of the time series with the highest correlation between different features and improve the generalization performance. Finally, the TC block utilizes TCN to extract high-level temporal features in the time series. The temporal features of different frequency bands are then concatenated and fed to the fully connected block for classification and identification.

Furthermore, this study implements the combination of Bi-FPN and CBAM modules. The ablation analysis reveals that both Bi-FPN and CBAM blocks contribute significantly to the performance of the BFATCNet model. In the BCI-2a dataset, CBAM improved the overall accuracy by 2.4%, BiFPN improved the overall accuracy by 8%, and the combination of BiFPN and CBAM improved the overall accuracy by 8.7%. In the BCI-2b dataset, CBAM improved the overall accuracy by 2.8%, BiFPN improved the overall accuracy by 10%, and the combination of BiFPN and CBAM improved the overall accuracy by 11.5%.

The proposed BFATCNet model surpasses state-of-the-art techniques for MI-EEG classification using the BCI-2a dataset and BCI-2b dataset, achieving accuracy of 87.5 and 86.3% for the subject-dependent, respectively. The model demonstrates an exceptional ability to extract MI features from raw EEG signals, without the need for data preprocessing operations such as artifact removal, major component extraction, and signal filtering. The augmentation of the BCI-2a dataset and BCI-2b dataset through data blending, signal scaling, and the addition of Gaussian noise contributes to improving the model's generalization ability and increasing its noise immunity. BFATCNet exhibits an overall improvement in EEG decoding for all MI categories and all subjects in the BCI-2a dataset and BCI-2b dataset, indicating its potential to learn universal EEG representations across categories and subjects.

However, our approach does have certain limitations. The model's performance evaluation is primarily based on the BCI-2a dataset and BCI-2b dataset, which may result in decreased performance when applied to other datasets. Although data augmentation has been applied to the BCI-2a dataset and BCI-2b dataset, the effectiveness of data augmentation may vary for different datasets or real-world data. Additionally, the complexity and computational requirements of the model may impose restrictions on its usage in real-time applications or embedded systems.

In future work, the proposed model can be further improved by researching and validating the effectiveness and applicability of data augmentation methods in different scenarios. Additionally, cross-modal deep learning models can be explored to integrate brainwave signals with other modalities such as functional magnetic resonance imaging (fMRI, eye-tracking data, text) to obtain a more comprehensive understanding of brain functionality. By combining multiple modalities, the interpretability and generalization of the model can be enhanced for brainwave signals. Alternatively, research can be conducted on reducing the computational complexity of the model to achieve higher performance and efficiency in real-time brain-computer interface applications.

## Data availability statement

The original contributions presented in the study are included in the article/supplementary material, further inquiries can be directed to the corresponding authors.

## Ethics statement

The studies involving humans were approved by Ethics Review Board of Shenzhen Academy of Robotics. The studies were conducted in accordance with the local legislation and institutional requirements. Written informed consent for participation was not required from the participants or the participants' legal guardians/next of kin in accordance with the national legislation and institutional requirements.

## Author contributions

XX: Conceptualization, Data curation, Software, Validation, Visualization, Writing – original draft. LC: Investigation, Writing – original draft, Writing – review & editing. SQ: Conceptualization, Data curation, Funding acquisition, Methodology, Project administration, Supervision, Writing – review & editing, Writing – original draft. FZ: Funding acquisition, Project administration, Writing – review & editing. XF: Methodology, Supervision, Writing – review & editing.
